# Type II NKT Cell Agonist, Sulfatide, Is an Effective Adjuvant for Oral Heat-Killed Cholera Vaccines

**DOI:** 10.3390/vaccines9060619

**Published:** 2021-06-08

**Authors:** Aqel Albutti, Stephanie Longet, Craig P. McEntee, Shauna Quinn, Alex Liddicoat, Cristiana Rîmniceanu, Nils Lycke, Lydia Lynch, Susanna Cardell, Ed C. Lavelle

**Affiliations:** 1 Adjuvant Research Group, School of Biochemistry and Immunology, Trinity Biomedical Sciences Institute, Trinity College Dublin, D02 R590 Dublin, Ireland; as.albutti@qu.edu.sa (A.A.); stephanie.longet@well.ox.ac.uk (S.L.); mcentec@tcd.ie (C.P.M.); squinn2@tcd.ie (S.Q.); LIDDICOA@tcd.ie (A.L.); lynchl3@tcd.ie (L.L.); 2 Department of Medical Biotechnology, College of Applied Medical Sciences, Qassim University, Buraydah 52571, Saudi Arabia; 3 Department of Microbiology and Immunology, Institute of Biomedicine, University of Gothenburg, Box 435, 405 30 Gothenburg, Sweden; cristiana.rimniceanu@gu.se (C.R.); nils.lycke@microbio.gu.se (N.L.); susanna.cardell@microbio.gu.se (S.C.); 4 Centre for Research on Adaptative Nanostructures and Nanodevices & Advanced Materials Bio-Engineering Research Centre, Trinity College Dublin, D02 PN40 Dublin, Ireland

**Keywords:** adjuvant, oral vaccine, NKT cells

## Abstract

Oral vaccination has the potential to offer a safer and more efficacious approach for protection against enteric pathogens than injection-based approaches, especially in developing countries. One key advantage is the potential to induce intestinal immune responses in addition to systemic immunity. In general, antigen delivery via the oral route triggers weak immune responses or immunological tolerance. The effectiveness of oral vaccination can be improved by co-administering adjuvants. However, a major challenge is the absence of potent and safe oral adjuvants for clinical application. Here, the Type II NKT cell activator sulfatide is shown for the first time to be an effective oral adjuvant for *Vibrio cholerae* vaccine antigens in a mouse model. Specifically, administration of sulfatide with the oral cholera vaccine Dukoral^®^ resulted in enhancement of intestinal antigen-specific IgA in addition to Th1 and Th17 immune responses. In summary, sulfatide is a promising adjuvant for inclusion in an oral cholera vaccine and our data further support the potential of adjuvants targeting NKT cells in new vaccine strategies.

## 1. Introduction

Oral vaccines are regarded as the optimal means to fight infections caused by intestinal pathogens as the oral route can facilitate the induction of both local and systemic immune responses [1]. Oral delivery is also the most convenient route of needle-free vaccine administration [2]. However, oral vaccines have to overcome difficult challenges linked to gastrointestinal biology, including degradation of fragile antigens in the acidic gastric environment and a requirement for vaccine design to promote mucosal immunity instead of tolerance [1,2]. Depending on the nature of the antigen, the addition of an adjuvant may be essential to overcome intestinal tolerance [2]. However, there are currently no licensed adjuvanted oral vaccines. Adjuvants targeting natural killer T (NKT) cells have been considered in the context of oral vaccine development as these cells are enriched at intestinal sites and have the potential to bridge innate and adaptive immunity [3]. We demonstrated that oral administration of the Type I NKT cell activator α-galactosylceramide (α-GalCer) enhanced intestinal and systemic antigen-specific humoral and cellular responses induced by experimental enterotoxigenic *Escherichia coli* [4], *Helicobacter pylori* [5] and *Vibrio cholerae* (*V. cholerae*) [6] antigens in mouse models. However, Type II NKT cells are more abundant in humans and could also be an attractive adjuvant target. These cells are reactive to endogenous or microbial glycolipids and phospholipids but α-GalCer or other α-linked glycolipids are not recognised by Type II NKT cells [7,8,9]. A sulfated glycosphingolipid (3-O-sulfogalactosylceramide) named ‘sulfatide’ was initially found in human brains in 1884 [10] but membranes of various tissues, for example, myelin of the central nervous system, gastrointestinal tract, pancreas, kidney and liver, are also enriched in sulfatide [9]. In the 2000s, sulfatide was identified as an activator of Type II NKT cells in mice [11] and a few years later, sulfatide CD1d-restricted NKT cells were also identified in the human intestinal mucosa [12]. Sulfatide was shown to be able to modulate immune responses in experimental mouse models of autoimmunity [13,14], tumour immunity [15] and viral [16] and bacterial [17] infections. Interestingly, some bacteria, such as enterotoxigenic *Escherichia coli* [18,19] and *Helicobacter pylori* [20], use sulfatide to adhere to intestinal mucosae.

For the first time, the potential of targeting intestinal Type II NKT cells using orally delivered sulfatide was investigated with whole-cell killed *V. cholerae* antigens and recombinant cholera toxin B (CTB) to assess the induction of antigen-specific humoral and cellular immune responses. Our results show that sulfatide is an effective oral adjuvant with *V. cholerae* antigens, enhancing intestinal immunoglobulin A (IgA), T helper 1 (Th1) and T helper 17 (Th17) responses in mice.

## 2. Materials and Methods

### 2.1. Animals

Nine- to sixteen-week-old female or male C57BL/6 mice were acquired from Charles River Laboratories. Male Tcrd^−/−^ mice knockout for the T cell receptor delta chain on a C57BL/6 background (Jackson Labs) were provided by Prof Lydia Lynch (Trinity College Dublin). Animals were housed in a specific pathogen-free environment. The work was approved by the TCD University Animal Research Ethics Committee (091210) and was carried out under HPRA license number AE19136/P079. CD1d-deficient (CD1d^−/−^) mice on a C57BL/6 background were bred at the Laboratory for Experimental Biomedicine, University of Gothenburg, and all mice were housed in microisolators for the duration of the study. All experiments were approved by the local ethics committee for animal experiments (Gothenburg, Sweden; ethical approval number 2070/19).

### 2.2. Immunisation of Mice

Groups of mice (*n* = 5–8) were immunised orally using 38.1 mm 18 g stainless steel curved feeding needles (Harvard Apparatus, Holliston, MA, USA) in three rounds on days 0 and 1; 14 and 15, and 28 and 29. One hour prior to immunisation, food was withdrawn. Each mouse was gavaged with 200 µL of solution prepared as follows: Sulfatide and α-GalCer stocks had been previously reconstituted in DMSO (1 mg/mL) (D2650, Sigma-Aldrich now Merck, Kenilworth, IL, USA) and frozen in small aliquots. Sulfatide (10, 20, 40 or 80 μg) (Matreya LCC, State College, PA, USA) or α-galactosylceramide (α-GalCer) (10 μg) (Avanti Lipids, Alabaster, AL, USA), as a positive control, was added to 100 µL of undiluted Dukoral^®^ (Valneva, Saint-Herblain, France). The final volume of solution was brought up to 200 μL with the addition of 0.3 M sodium bicarbonate buffer (pH 9). The group ‘Dukoral^®^ alone’ received the same solution without any adjuvant. The naïve control group received phosphate-buffered saline (PBS) instead of Dukoral^®^. Faecal pellet samples were collected on day 42 and organs/tissues or blood samples were collected on day 43.

### 2.3. Isolation of Splenocytes and Mesenteric Lymph Node Cells

Mice were sacrificed by cervical dislocation. Spleens and mesenteric lymph nodes (MLNs) were harvested. Tissues were processed through 70 μm nylon cell strainers (BD Falcon) with complete Roswell Park Memorial Institute (RPMI) 1640 medium (Biosera): RPMI supplemented with 2 mM L-glutamine (Gibco now Thermo Fisher Scientific, Waltham, MA, USA), 50 U/mL penicillin (Gibco), 50 μg/mL streptomycin (Gibco) and 8% (*v*/*v*) inactivated foetal bovine serum (FBS) (Biosera, San Diego, CA, USA). Subsequently, the cells were subjected to 5 min of centrifugation at 1200× *g* rpm and splenocyte pellets were resuspended for 2 min in 1 mL 0.08% ammonium chloride to lyse red blood cells. Complete RPMI was used to wash cells before being subjected once again to centrifugation. MLN preparations were resuspended in 1ml and splenocytes in 5 mL of T cell medium: RPMI containing 0.88 mM L-glutamine (Gibco), 0.88 mM sodium pyruvate (Gibco), 0.04 mM β-mercaptoethanol, 0.88% (*v*/*v*) MEM non-essential amino acids (Gibco), 0.35% (*v*/*v*) 100X MEM vitamins (Gibco), 4.4 U/mL penicillin (Gibco), 4.4 μg/mL streptomycin (Gibco) and 10% (*v*/*v*) inactivated FBS (Biosera), prior to being counted. Splenocytes and MLN cells were plated at 2 × 10^6^ cells/mL in 200 μL T cell medium and 1 × 10^6^ cells/mL in 200 μL T cell medium, respectively, in 96-well round bottom tissue culture plates (Greiner, Kremsmünster, Austria).

### 2.4. Cytokine Quantification by Enzyme-Linked Immunosorbent Assay (ELISA)

Splenocytes and MLN cells were restimulated ex vivo with 1.6 × 10^7^ and 1.6 × 10^8^ bacteria/mL from Dukoral^®^, respectively. To eliminate recombinant CTB from Dukoral^®^, the preparation was washed three times with sterile PBS 1× before using it for restimulation of cells. Isolated cells were cultured at 37 °C with an atmospheric condition of 5% CO_2_ with 95% humidity. Following a 72 h incubation, supernatants were stored at −20 °C until required for analysis by ELISA. Interferon γ (IFNγ) (R&D systems) and interleukin 17A (IL-17A) (Biolegend, San Diego, CA, USA) were used as per manufacturer’s instructions. Raw data can be found in Table S1.

### 2.5. Collection of Faecal and Serum Samples

Faecal pellet supernatants and serum samples were collected and prepared as previously described [4,6]. Mice were placed into individual cages and 5 fresh faecal pellets were collected in 500 µL of cold faecal pellet buffer (0.1 mg/mL soybean trypsin inhibitor (STI) (Sigma-Aldrich), 1% bovine serum albumin (BSA), 25 mM ethylenediaminetetraacetic acid (EDTA) (Gibco), 1 mM PEFABloc (Sigma Aldrich now Merck, Kenilworth, IL, USA), 50% glycerol in 1× PBS)) and kept on ice for 4 h. Samples were then weighed, emulsified and centrifuged at 15,400× *g* 5 min at 4 °C and supernatants were stored at −20 °C until further use. The weight of faecal pellets was used to normalise the results. Blood was obtained from mice by an incision in the tail vein. Samples were left to coagulate overnight at 4 °C and then centrifuged at 9200× *g* 10 min. The serum was stored at −20 °C until further use.

### 2.6. Preparation of Intestinal Tissues

Intestinal tissues were collected using the perfusion–extraction (PERFEXT) method as previously described [4,6,21]. After sacrifice, mice were perfused with 20 mL 0.1% heparin sulphate (Sigma-Aldrich)—PBS through the heart and the caudal mesenteric arteries. Intestines were washed in PBS and were placed in 270 μL ice cold sample buffer (0.1 mg/mL STI, 0.05 M EDTA, 1 mM PEFABloc, 0.1% BSA, 0.05% Tween20 in 1× PBS). Thirty microliters of 20% saponin from quillaja bark (SigmaAldrich) were added into each tube and incubated overnight at 4 °C. Supernatants were isolated following centrifugation at 14,000× *g* 10 min at 4 °C and stored at −20 °C until further use.

### 2.7. Measurement of Whole Bacteria-Specific Antibodies by ELISA

Bacterium-specific IgA in faecal pellets and intestinal tissues and IgG, IgG1, IgG2b and IgG2c titres were determined in serum. Plates were coated with 50 μL per well of 4 × 10^6^/well killed *Vibrio cholerae* from the Dukoral^®^ vaccine, which were diluted in 1× PBS overnight at 4 °C in 96-well medium-binding ELISA plates (Greiner Bio-One). ELISA plates were washed in 1× PBS–Tween 0.05% (Sigma) three times and blocked with 200 μL 0.1% BSA (Fisher Bioreagents, Pittsburgh, PA, USA) for an hour at a temperature of 37 °C. The plates were flicked thoroughly to discharge the blocking solution and tapped dry. Samples were serially diluted in 0.1% BSA–PBS–Tween before being applied to the plates in a volume of 50 μL per well and incubated overnight at 4 °C. Plates were washed 3 times. To each well, 50 μL of detection antibody was added: anti-IgA coupled to horseradish peroxidase (HRP) (Southern Biotech, Birmingham, AL, USA, 0.5 μg/mL), anti-IgG (BD Pharmingen, San Diego, CA, USA, 3μg/mL), anti-IgG1 (BD Pharmingen, 0.1 μg/mL), biotinylated IgG2b (BD Pharmingen, 0.1 μg/mL) or anti-IgG2c coupled to HRP (Bio-Rad, Hercules, CA, USA, 0.1 μg/mL), before plates were incubated for 1 h at RT. Following the incubation with biotinylated IgG/IgG1/IgG2b antibodies, plates were washed 3 times and 50 μL/well of 700 ng/mL HRP-conjugated streptavidin (BD 554066) was added and plates were placed in the dark at room temperature for 30 min. Finally, plates were washed 4 times and 1 mg/mL O-phenylenediamine dihydrochloride substrate (Sigma-Aldrich, now Merck, Kenilworth, IL, USA) was prepared in 0.1 M phosphate citrate buffer (pH 5) containing 4 μL H_2_O_2_ per 10 mL substrate and 50 μL were added per well. The plates were left to develop at RT and the reaction was then stopped by the addition of 25 μL/well of 1 M H_2_SO_4_, then the absorbance at 492 nm was read using a microplate reader (Thermo Scientific) running Scan-IT software (Thermo Fisher Scientific, Waltham, MA, USA) to acquire data. For each sample, an endpoint titre was defined as the reciprocal of the highest sample dilution that gave a reading (OD) above the cut-off. The cut-off was determined for each experimental group as the mean OD + 2 standard deviations of control samples [22]. Raw data can be found in Table S1.

### 2.8. Measurement of CTB-Specific Antibodies by ELISA

CTB-specific IgA in faecal pellets and intestinal tissues as well as IgG, IgG1, IgG2b and IgG2c titres were determined in serum. Each well in 96-well medium-binding ELISA plates (Grenier Bio-One) was coated with 50 μL of 0.3 nmol/mL of GM1 ganglioside diluted in 1× PBS and incubated overnight at 4 °C. ELISA plates were washed with 1× PBS-tween 0.05% (Sigma) three times and blocked with 200 μL 0.1% bovine serum albumin (BSA) (Fisher) for an hour at 37 °C. The plates were flicked thoroughly to discharge the blocking solution and tapped dry. Samples were serially diluted in 0.1% BSA–PBS–Tween before being applied to the plates in a volume of 50 μL per well and incubated overnight at 4 °C. Plates were washed 3 times. To each well, 50 μL of detection antibody was added: anti-IgA coupled to HRP (Southern Biotech, 0.5 μg/mL), anti-IgG (BD Pharmingen, 3μg/mL), anti-IgG1 (BD Pharmingen, 0.1 μg/mL), biotinylated IgG2b (BD Pharmingen, 0.1 μg/mL) or anti-IgG2c coupled to HRP (Bio-Rad, 0.1 μg/mL), before plates were incubated for 1 h at RT. Following the incubation with biotinylated IgG/IgG1/IgG2b antibodies, plates were washed 3 times and 50 μL/well of 700 ng/mL HRP-conjugated streptavidin (BD 554066) was added and plates were placed in the dark at room temperature for 30 min. Finally, plates were washed 4 times and 1 mg/mL O-phenylenediamine dihydrochloride substrate (Sigma-Aldrich) was prepared in 0.1 M phosphate citrate buffer (pH 5) containing 4 μL H_2_O_2_ per 10 mL substrate and 50 μL were added per well. The plates were left to develop at RT and the reaction was then stopped by the addition of 25 μL/well of 1 M H_2_SO_4_, then the absorbance at 492 nm was read using a microplate reader (Thermo Scientific) running Scan-IT software (Thermo Scientific) to acquire data. For each sample, an endpoint titre was defined as the reciprocal of the highest sample dilution that gave a reading (OD) above the cut-off, which was determined for each experimental group as the mean OD + 2 standard deviations of control samples. Raw data can be found in Table S1.

### 2.9. Statistical Analysis

GraphPad Prism 7 software was used for statistical analyses. For the antibody analyses, one-way analysis of variance was used to determine significant differences between Dukoral^®^ alone and Dukoral^®^ co-administered with an adjuvant. The degree of significance was calculated by Dunnett’s or Tukey’s multiple comparisons test. For the IFNγ and IL-17A analyses, a Kruskal–Wallis test was used to determine significant differences between Dukoral^®^ alone and Dukoral^®^ co-administered with an adjuvant. The degree of significance was calculated by Dunn’s multiple comparisons test.

## 3. Results

### 3.1. Oral Co-Administration of Sulfatide with Dukoral^®^ Enhanced CTB- and Bacterium-Specific Intestinal IgA Responses Compared to Vaccination with Dukoral^®^ Alone

**Figure 1:**
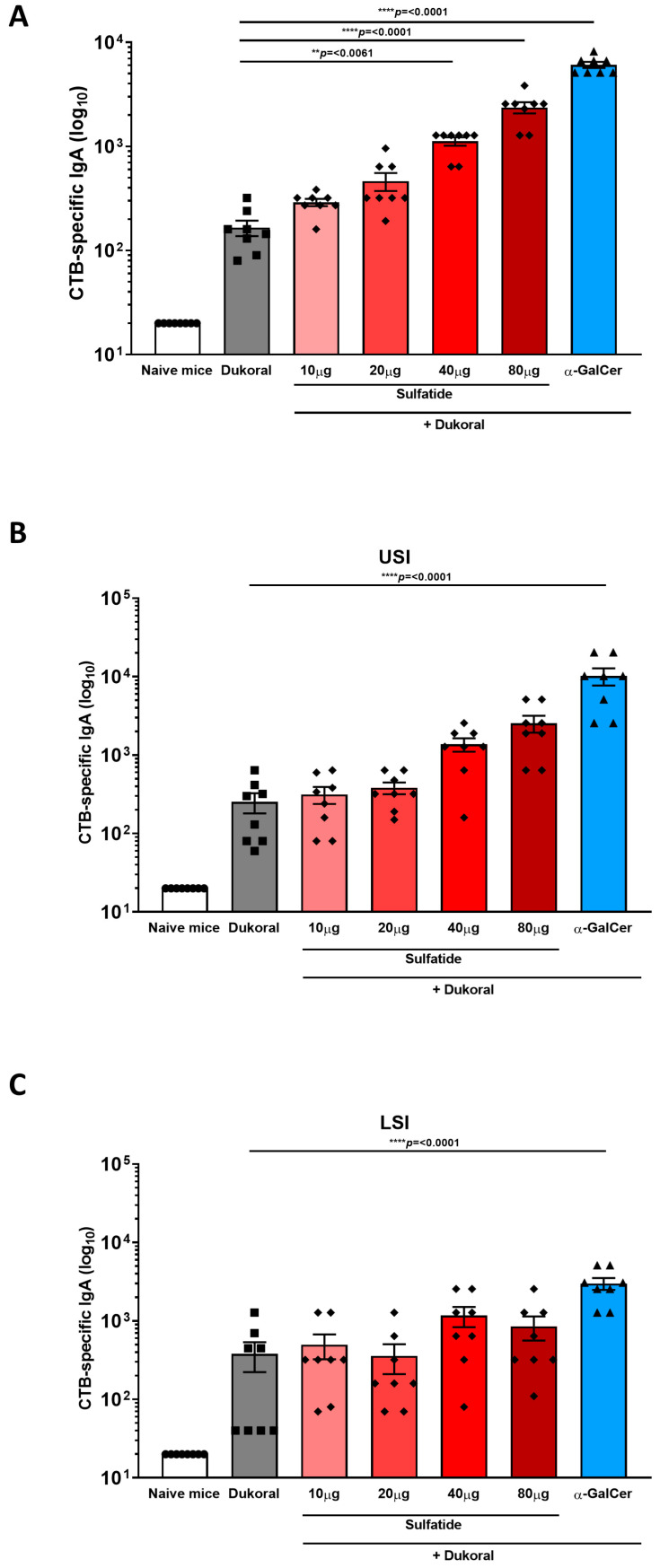
Sulfatide enhances intestinal cholera toxin subunit B-specific IgA responses following oral co-administration with Dukoral^®^. Female C57BL/6 mice were immunised orally with Dukoral^®^ co-administered with 10, 20, 40 or 80 μg sulfatide, or 10 µg α-GalCer in comparison with mice immunised with Dukoral^®^ alone or with PBS (naïve mice). On day 42 and 43, faecal pellets and intestinal tissues were collected, respectively. Endpoint titres of CTB-specific IgA in faecal pellets (**A**), upper small intestine (USI) (**B**) and lower small intestine (LSI) (**C**) were determined by ELISA. Results presented are mean titres ±SEM. Significant differences between the group of mice vaccinated with Dukoral^®^ alone and Dukoral^®^ co-administered with an oral adjuvant are shown. Symbols show the groups of mice. Circles: naïve mice. Squares: mice immunised with Dukoral^®^ alone. Rhombus: mice immunised with Dukoral^®^ and sulfatide. Triangles: mice immunised with Dukoral^®^ and α-GalCer. Each symbol represents one animal in the group.

**Figure 2:**
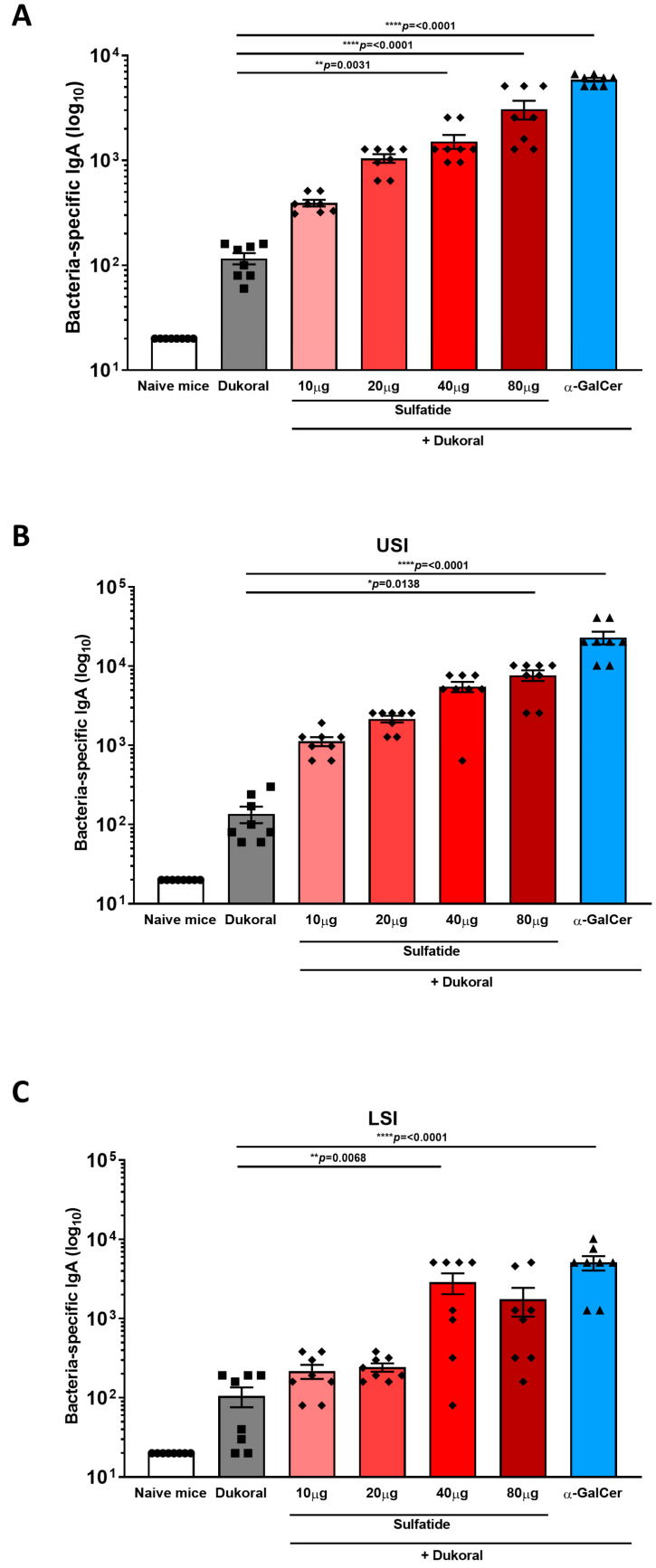
Sulfatide enhances intestinal *Vibrio cholerae*-specific IgA responses following oral co-administration with Dukoral^®^. Female C57BL/6 mice were immunised orally with Dukoral^®^ co-administered with 10, 20, 40 or 80 μg sulfatide, or 10 µg α-GalCer in comparison with mice immunised with Dukoral^®^ alone or with PBS (naïve mice). On day 42 and 43, faecal pellets and intestinal tissues were collected, respectively. Endpoint titres of bacterium-specific IgA in faecal pellets (**A**), upper small intestine (USI) (**B**) and lower small intestine (LSI) (**C**) were determined by ELISA. Results presented are mean titres ±SEM. Significant differences between the group of mice vaccinated with Dukoral^®^ alone and Dukoral^®^ co-administered with an oral adjuvant are shown. Symbols show the groups of mice. Circles: naïve mice. Squares: mice immunised with Dukoral^®^ alone. Rhombus: mice immunised with Dukoral^®^ and sulfatide. Triangles: mice immunised with Dukoral^®^ and α-GalCer. Each symbol represents one animal in the group.

Antigen-specific humoral immunity in the intestinal tract is dominated by IgA, thus, determining the induction of antigen-specific IgA antibody titres in faecal pellets and intestinal tissues can provide an excellent measure of the efficiency of a vaccine formulations. Female C57BL/6 mice were immunised orally with Dukoral^®^ and 10, 20, 40 or 80 µg sulfatide or with Dukoral^®^ alone in three rounds on days 0 and 1; 14 and 15; and 28 and 29. We previously demonstrated that the iNKT cell activator α-GalCer was an effective adjuvant to enhance intestinal antigen-specific IgA responses following oral co-administration of α-GalCer with Dukoral^®^ [6], thus, a group of mice immunised with Dukoral^®^ and 10 µg α-GalCer was included as a positive control in this study. As Dukoral^®^ consists of inactivated of *Vibrio cholerae* (*V. cholerae*) O1 Inaba and Ogawa as well as a recombinant CTB subunit, both CTB- and bacterium-specific IgA responses were measured by ELISA in faecal pellets and intestinal tissues were collected two weeks following the third round of immunisation. A dose-dependent increase in CTB-specific IgA antibody titres was measured in faecal pellets from mice given sulfatide together with Dukoral^®^ in comparison with mice vaccinated with Dukoral^®^ alone (Figure 1A). IgA responses in the group having received the highest doses of sulfatide (40 and 80 µg) with Dukoral^®^ were significantly higher than in the group which received Dukoral^®^ alone and were not significantly different from the positive control group which received Dukoral^®^ with α-GalCer. Higher antigen-specific IgA responses were also observed in upper small intestinal tissue (USI) in the group of mice immunised with Dukoral^®^ and 80 µg sulfatide in comparison with Dukoral^®^ alone (Figure 1B), while a similar trend was observed in the lower small intestine (LSI) (Figure 1C). A dose-dependent increase in bacterium-specific IgA responses was also measured in the faecal pellets (Figure 2A), USI (Figure 2B) and LSI (Figure 2C) of mice which received Dukoral^®^ and sulfatide in comparison with mice immunised with Dukoral^®^ alone. Bacterium-specific IgA responses in the group which received Dukoral^®^ and the highest doses of sulfatide (40 and 80 µg) were significantly higher than in the group immunised with Dukoral^®^ alone in faecal pellets (Figure 2A). A significantly higher IgA response was also observed in mice vaccinated with Dukoral^®^ and 80 µg sulfatide in the USI (Figure 2B) and 40 μg sulfatide in the LSI (Figure 2C).

Overall, these results show that oral vaccination with Dukoral^®^ and 40 or 80 μg sulfatide increased antigen-specific IgA responses in the intestinal lumen and intestinal tissues.

### 3.2. Oral Co-Administration of Sulfatide with Dukoral^®^ Enhanced Mucosal IFNγ and IL-17A Responses, as Well as Systemic IgG Responses

**Figure 3:**
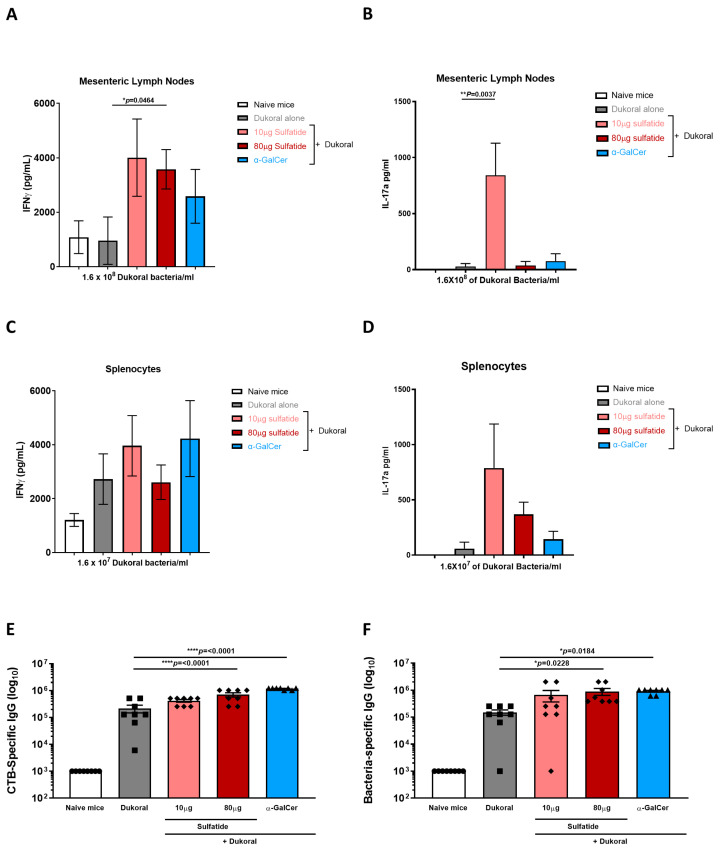
Oral co-administration of Dukoral^®^ with sulfatide enhances local and systemic IFNγ and IL-17A responses and antigen-specific serum IgG responses. Female C57BL/6 mice were immunised orally with Dukoral^®^ co-administered with 10 or 80 µg sulfatide or 10 µg α-GalCer or with Dukoral^®^ alone or with PBS (naïve mice). On day 43, spleens, MLNs and blood were collected. MLN cells (**A**,**B**) and splenocytes (**C**,**D**) were restimulated with 1.6 × 10^8^ and 1.6 × 10^7^ Dukoral^®^ bacteria/mL, respectively, for 72 h before measuring IFNγ (**A**,**C**) and IL-17A (**B**,**D**) in the supernatant by ELISA. Endpoint titres of CTB-specific IgG (**E**) and bacterium-specific IgG (**F**) were determined in serum by ELISA. Results presented are mean concentrations (pg/mL) or titres ±SEM. Significant differences between the group vaccinated with Dukoral^®^ alone or Dukoral^®^ and sulfatide are shown. Symbols show the groups of mice. Circles: naïve mice. Squares: mice immunised with Dukoral^®^ alone. Rhombus: mice immunised with Dukoral^®^ and sulfatide. Triangles: mice immunised with Dukoral^®^ and α-GalCer. Each symbol represents one animal in the group.

Even though antibody responses are essential in protection against cholera [23,24], it was shown that T cell responses were also induced following cholera infections in humans [25,26]. CD4^−/−^ mice did not respond with antigen-specific intestinal IgA following oral immunisation with cholera toxin, highlighting the crucial role of CD4 T cells in induction of protective IgA responses [27]. More specifically, IFNγ-R signalling was reported to promote antigen-specific IgA responses in a mouse model [28] and IL-17 was detected following restimulation of whole blood from patients with a *Vibrio cholerae* membrane preparation. However, similar results were not observed following oral administration of Dukoral^®^ [29]. A better priming of Th1 and Th17 responses, leading to long lasting protection, might be reached with the addition of an oral adjuvant with Dukoral^®^. In this study, the benefits of using sulfatide as an adjuvant to promote Dukoral^®^-specific T cell-mediated responses were evaluated. To compare the efficacy of high and low doses of sulfatide as an adjuvant to induce Th1 and Th17 responses, female C57BL/6 mice were immunised orally with Dukoral^®^ and 10 or 80 µg sulfatide or with Dukoral^®^ alone or Dukoral^®^ and 10 µg α-GalCer using the scheme of immunisation previously described. Two weeks following the last round of immunisation, mice were sacrificed. Mesenteric lymph nodes (MLNs) and spleens were processed. MLN cells and splenocytes were restimulated with 1.6 × 10^8^ and 1.6 × 10^7^ Dukoral^®^ bacteria/mL, respectively. Following a 72-h incubation, IFNγ and IL-17A were measured in the supernatant by ELISA. A significantly higher antigen-specific MLN IFNγ response was detected in mice immunised with Dukoral^®^ and 80 μg sulfatide than in MLN from mice immunised with Dukoral^®^ alone (Figure 3A). Interestingly, a significantly higher release of IL-17A was also measured in the supernatant of MLN cells from mice immunised with Dukoral^®^ and 10 μg sulfatide compared to MLN cells from mice vaccinated with Dukoral^®^ alone (Figure 3B). As previously observed in other vaccination studies, α-GalCer enhanced antigen-specific IFNγ but not IL-17A responses in MLNs (Figure 3A,B) [5,6]. Restimulated splenocytes from mice immunised with Dukoral^®^ and 10 μg sulfatide or α-GalCer showed a trend towards higher release of IFNγ compared to the mice immunised with Dukoral^®^ alone (Figure 3C). A higher release of IL-17A was also measured in the supernatant of splenocytes from mice vaccinated with Dukoral^®^ co-administered with 10 and 80 μg sulfatide, especially with the lowest dose of sulfatide (Figure 3D), which correlates with the IL-17A response determined in MLNs (Figure 3B). However, none of these differences observed in the spleen reached significance. As previously observed in MLNs, vaccination with Dukoral^®^ and α-GalCer generated an antigen-specific IFNγ response but not an IL-17A response (Figure 3C,D).

In order to further analyse systemic immune responses, we evaluated whether sulfatide was able to enhance antigen-specific IgG responses. Blood was collected two weeks after the final vaccination. Endpoint titres of CTB- and bacterium-specific IgG responses were determined in sera. Oral vaccination with Dukoral^®^ alone triggered high CTB- (Figure 3E) and bacterium-specific (Figure 3F) IgG responses compared to naïve mice but these responses were significantly enhanced by co-administration of 80 μg sulfatide or α-GalCer. To determine the profile of IgG responses, CTB- and bacterium-specific IgG1 (Figure S1A,B), IgG2b (Figure S1C,D) and IgG2c (Figure S1E,F) subclasses were also measured. A predominant profile was observed for CTB-specific IgG1 responses with 80 μg sulfatide and for CTB-specific IgG2c with 10 μg sulfatide. IgG2c is an IFNγ-dependent-isotype and, interestingly, these findings correlate with the trend of IFNγ release observed in the spleen (Figure 3C).

The sum of these results suggests that sulfatide is an effective oral adjuvant for promoting mucosal T cell responses in addition to circulating IgG antibodies.

### 3.3. The Enhancement of Antigen-Specific Intestinal IgA Response by Sulfatide Was Reduced in CD1d^−/−^ Mice but Not in γδ T Cell-Deficient Mice

**Figure 4:**
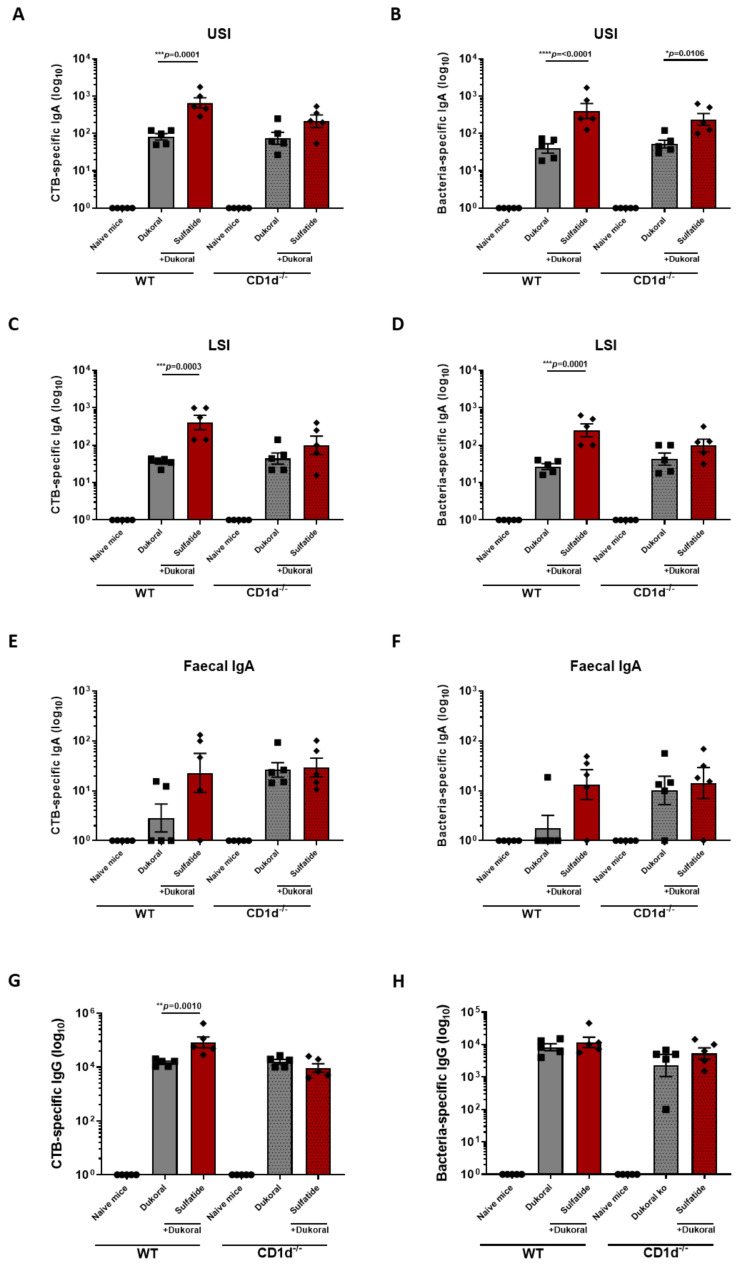
The enhancement of antigen-specific IgA and IgG responses by sulfatide is reduced in CD1d^−/−^ mice. Female C57BL/6 WT and CD1d^−/−^ mice were immunised orally with Dukoral^®^ co-administered with 80 µg sulfatide in comparison with mice immunised with Dukoral^®^ alone or with PBS (naïve mice). Two weeks following the last round of immunisation, intestinal tissues, faecal pellets and blood were collected. Endpoint titres of CTB-specific IgA in upper small intestine (USI) (**A**), LSI (**C**), faecal pellets (**E**) and CTB-specific IgG in serum (**G**) were determined by ELISA. Similar analysis was performed to determine endpoint titres of bacteria-specific IgA in upper small intestine (USI) (**B**), LSI (**D**), faecal pellets (**F**) and CTB-specific IgG in serum (**H**). Results presented are mean titres ±SEM. Significant differences between the group of mice vaccinated with Dukoral^®^ alone and Dukoral^®^ co-administered with sulfatide are shown. Symbols show the groups of mice. Circles: naïve mice. Squares: mice immunised with Dukoral^®^ alone. Rhombus: mice immunised with Dukoral^®^ and sulfatide. Each symbol represents one animal in the group.

NKT cells are defined as T cells that recognise antigens presented by class I MHC-like molecules such as CD1d [30]. To assess the dependence of sulfatide-driven responses on CD1d, female C57BL/6 CD1d^−/−^ mice and WT mice were immunised orally with Dukoral^®^ and the highest dose of sulfatide tested in this study (80 µg) or Dukoral^®^ alone. Antigen-specific IgA responses were measured in intestinal tissues and faecal pellets. As previously observed, CTB- and bacterium-specific IgA responses in the USI (Figure 4A,B) and LSI (Figure 4C,D) were significantly increased in WT mice immunised with Dukoral^®^ and sulfatide compared to WT mice vaccinated with Dukoral^®^ alone. The significant difference in CTB- and bacterium-specific IgA responses between mice immunised with Dukoral^®^ and sulfatide and the mice immunised with Dukoral^®^ alone was reduced in the USI (Figure 4A,B) and LSI (Figure 4C,D) of CD1d^−/−^ mice. Enhanced CTB- and bacterium-specific IgA responses were confirmed in faecal pellets of WT mice immunised with Dukoral^®^ in combination with sulfatide compared to the WT mice vaccinated with Dukoral^®^ alone. Surprisingly, both CTB- and bacterium-specific IgA responses in faecal pellets of CD1d^−/−^ mice immunised with Dukoral^®^ alone were enhanced compared to the WT mice which received the vaccine alone (Figure 4E,F). However, an increase in IgA responses in CD1d^−/−^ mice immunised with Dukoral^®^ and sulfatide was not observed. A significant enhancement of CTB–IgG responses was determined in WT mice immunised with Dukoral^®^ and sulfatide compared to the WT mice vaccinated with Dukoral^®^ alone. This significant difference between these groups of mice was lost in CD1d^−/−^ mice (Figure 4G). These results suggest a role of CD1d in enhancement of antigen-specific antibody responses by sulfatide.

Several studies have found that sulfatide-reactive CD1d-restricted T cells also expressed the γδT cell receptor in humans [31,32] and mice [33]. To investigate whether the immune responses to the vaccination with sulfatide were dependent on γδT cells in our model, male Tcrd^−/−^ C57BL/6 mice were immunised orally with Dukoral^®^ and 80 µg of sulfatide and described in comparison with male WT C57BL/6 mice. Two weeks following the last round of immunisation, antigen-specific IgA was analysed in intestinal tissues and faecal pellets, as well as antigen-specific IgG in serum. As previously observed in female WT mice, we measured an enhancement of CTB- and bacterium-specific IgA responses in USI and LSI intestines in WT mice immunised with Dukoral^®^ and sulfatide compared to the WT mice vaccinated with Dukoral^®^ alone (Figure S2A–D). A similar enhancement was observed in Tcrd^−/−^ mice which received the vaccine and sulfatide (Figure S2A–D). CTB- and bacterium-specific IgA responses were also measured in faecal pellets. The difference of CTB- and bacterium-specific IgA responses observed between the WT mice immunised with Dukoral^®^ and sulfatide and Dukoral^®^ alone was less marked in male WT mice than those previously observed in female WT mice. However, there was no major difference between the sulfatide groups in WT or Tcrd^−/−^ mice in terms of antigen-specific IgA responses (Figure S2E,F). Finally, CTB- and bacterium-specific IgG responses were determined in serum. Similar increases in CTB- and bacterium-specific IgG responses were measured in both WT and Tcrd^−/−^ mice immunised with sulfatide and Dukoral^®^ vaccine (Figure S2G,H). These results show that antigen-specific IgA and IgG responses to Dukoral^®^ given in conjunction with sulfatide are not affected by the deficiency of γδT cells, suggesting that these cells do not mediate enhanced immune responses following vaccination with Dukoral^®^ and sulfatide.

## 4. Discussion

This study demonstrates that the Type II NKT cell activator sulfatide is an effective adjuvant in an oral cholera vaccine. We observed an enhancement of antigen-specific IgA responses in the intestinal lumen and tissues, suggesting an increase in local production of IgA by sulfatide. We also determined an enhancement in antigen-specific IFNγ and IL-17A responses in the MLNs. It is known that Type II NKT cells can influence systemic IgG and IgM responses [34]. However, to our knowledge, this is the first study showing an impact of sulfatide on intestinal antigen-specific IgA and cellular immune responses. Interestingly, we observed that the highest dose of sulfatide (80 μg) was most efficient in enhancing IgA responses, whereas the lowest dose (10 μg) could induce higher cellular responses. Surprisingly, IL-17A was only detected in supernatants from MLN cells of mice immunised with Dukoral^®^ and 10 μg sulfatide, whereas the highest intestinal IgA response was observed in mice vaccinated with Dukoral^®^ and 80 μg sulfatide. Hirota and colleagues demonstrated that local Th17 cells were crucial to induce intestinal T cell-dependent IgA production [35] while Cao et al. found that IL-17A induced pIgR expression on the intestinal epithelium and enhanced transepithelial IgA secretion [36]. Datta et al. showed that IL-17 was required for the induction of cell-mediated immunity and antigen-specific serum and mucosal antibody responses following oral immunisation with ovalbumin and CT in mice [37]. In contrast, we previously demonstrated that IL-17R signalling was not required for the ability of α-GalCer to enhance intestinal antigen-specific IgA responses, including in the context of oral cholera vaccines [5,6]. In the present study, our results suggest an ability of sulfatide to enhance antigen-specific IL-17A responses but do not show any correlation between intestinal IgA responses and the magnitude of the antigen-specific IL-17A response. Sulfatide may use an IL-17-independent pathway for enhancing antigen-specific IgA in intestinal tissues and IgA secretion. We previously showed that α-GalCer enhanced Dukoral^®^-specific serum IgG and systemic cellular responses [6]. In this study, we showed a dose-dependent increase in systemic antigen-specific IgG responses by sulfatide even though Dukoral^®^ alone was able to induce high IgG response in serum. Regarding systemic IFNγ and IL-17A responses, a trend towards higher splenic antigen-specific IFNγ and IL-17A responses was measured, especially in mice immunised with Dukoral^®^ co-administered with 10 μg sulfatide.

The basis for differential induction of mucosal humoral and cellular immunity by co-administration of different doses of sulfatide should be further elucidated. It was suggested that the failure of the current oral cholera vaccines to activate CD4^+^ responses might be linked to the short duration of protection [29]. The use of an oral adjuvant such as sulfatide might be a way to improve long term immunity of cholera vaccines. In this study, we demonstrated a dependence of the oral adjuvanticity of sulfatide on CD1d. Unexpectedly, we observed a higher faecal IgA response in CD1d^−/−^ mice compared to WT mice immunised with Dukoral^®^ alone. This might be due to a crosstalk between NKT cells and intestinal microbiota. Indeed, NKT cells influence bacterial colonisation and the composition of the intestinal microbiota. This could influence the response to Dukoral^®^ [38,39]. However, sulfatide did not increase the antigen-specific IgA response in faecal pellets of CD1d^−/−^ mice compared to WT mice, which is consistent with our observations in intestinal tissues and serum. Even though some studies reported CD1d-mediated sulfatide recognition by γδT cells in intestinal epithelium and peripheral blood [32], we did not detect any involvement of γδT cells in enhancement of intestinal and systemic antibody responses induced by sulfatide in our model.

## 5. Conclusions

The results of this study provide the first evidence that sulfatide is effective as an oral vaccine adjuvant in mice. This suggests that Type II NKT cell may provide a novel target in developing improved oral vaccines. However, to our knowledge, exogenous sulfatide has been never administered to humans. Type II NKT cells can be divided into anti- and pro-inflammatory subsets [40]. In rodent models, the activation of Type II NKT cells can inhibit [13,14] or exacerbate some inflammatory conditions [41,42]. Careful optimisation of doses should be carried out to tailor immune responses and avoid detrimental inflammatory responses. In addition, sulfatide was shown to be presented by CD1a, CD1b and CD1c to T cells clones derived from human PBMCs [43]. Mice only express CD1d but it is crucial to determine whether sulfatide presentation by other CD1 molecules can be detected at the intestinal level in humans and how it might impact on induction of local immune responses. Our results support further evaluation of the efficacy and safety of NKT cell-targeting mucosal vaccine adjuvants.

## Data Availability

The data presented in this study are available within the article. Rough data are available on request from the corresponding author.

## Supplementary Materials

The following are available online at https://www.mdpi.com/article/10.3390/vaccines9060619/s1, Figure S1: Antigen-specific IgG1, IgG2b and IgG2c responses in serum of mice following immunisation with Dukoral^®^ and sulfatide; Figure S2: A similar increase in antigen-specific IgA and IgG responses is measured in WT and Tcrd^−/−^ mice following oral co-administration with Dukoral^®^ and sulfatide; Table S1: Raw data.
