# Supplementary material for: Type II NKT Cell Agonist, Sulfatide, Is an Effective Adjuvant for Oral Heat-Killed Cholera Vaccines
Source: Vaccines (Basel). 2021 Jun 8;9(6):619. doi: 10.3390/vaccines9060619 (PMC8230052; doi:10.3390/vaccines9060619)
Supplement: Supplementary file 1 [file vaccines-09-00619-s001.zip › vaccines-1226120 sup proof done.pdf]

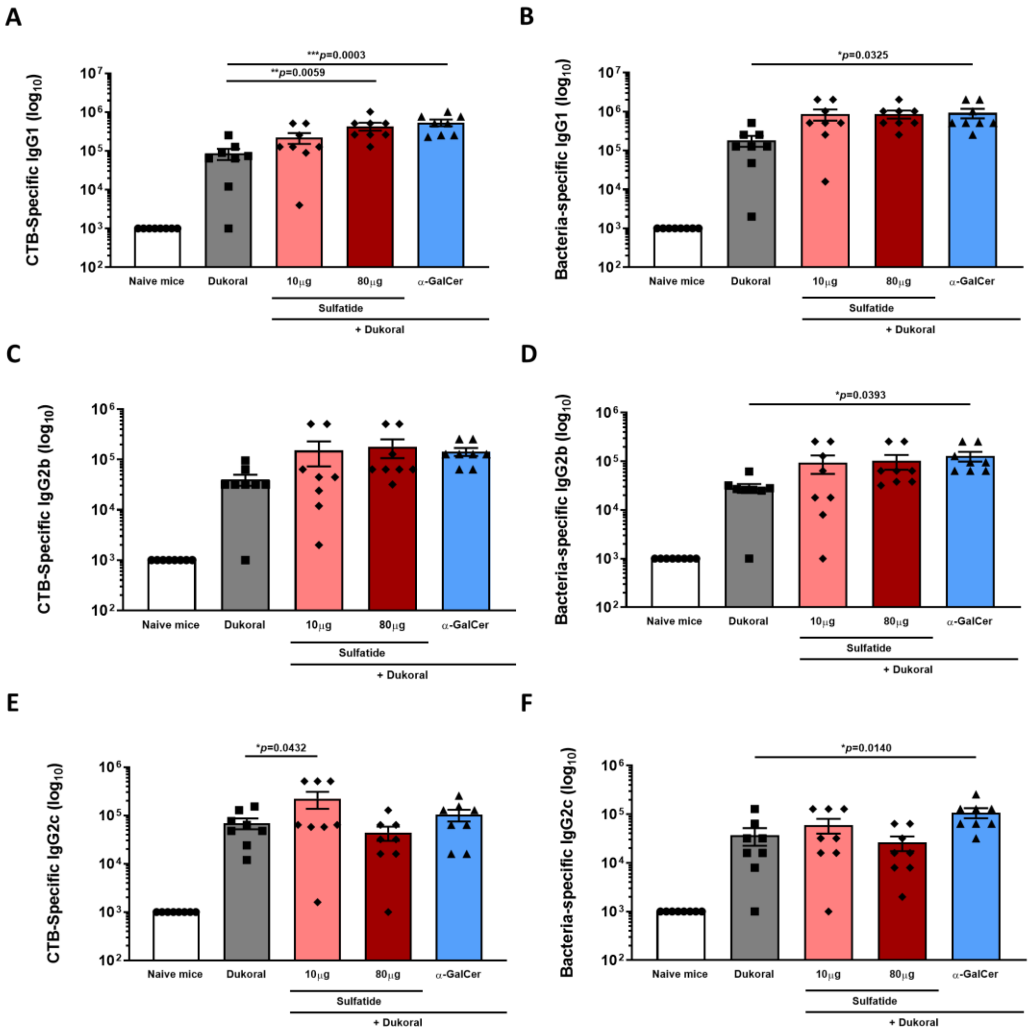

Figure S1. Antigen-specific IgG1, IgG2b and IgG2c responses in serum of mice following immunisation with Dukoral® and sulfatide. Symbols show the groups of mice. Circles: naïve mice. Squares: mice immunised with Dukoral® alone. Rhombus: mice immunised with Dukoral® and sulfatide. Triangles: mice immunised with Dukoral® and α-GalCer. Each symbol represents one animal in the group.

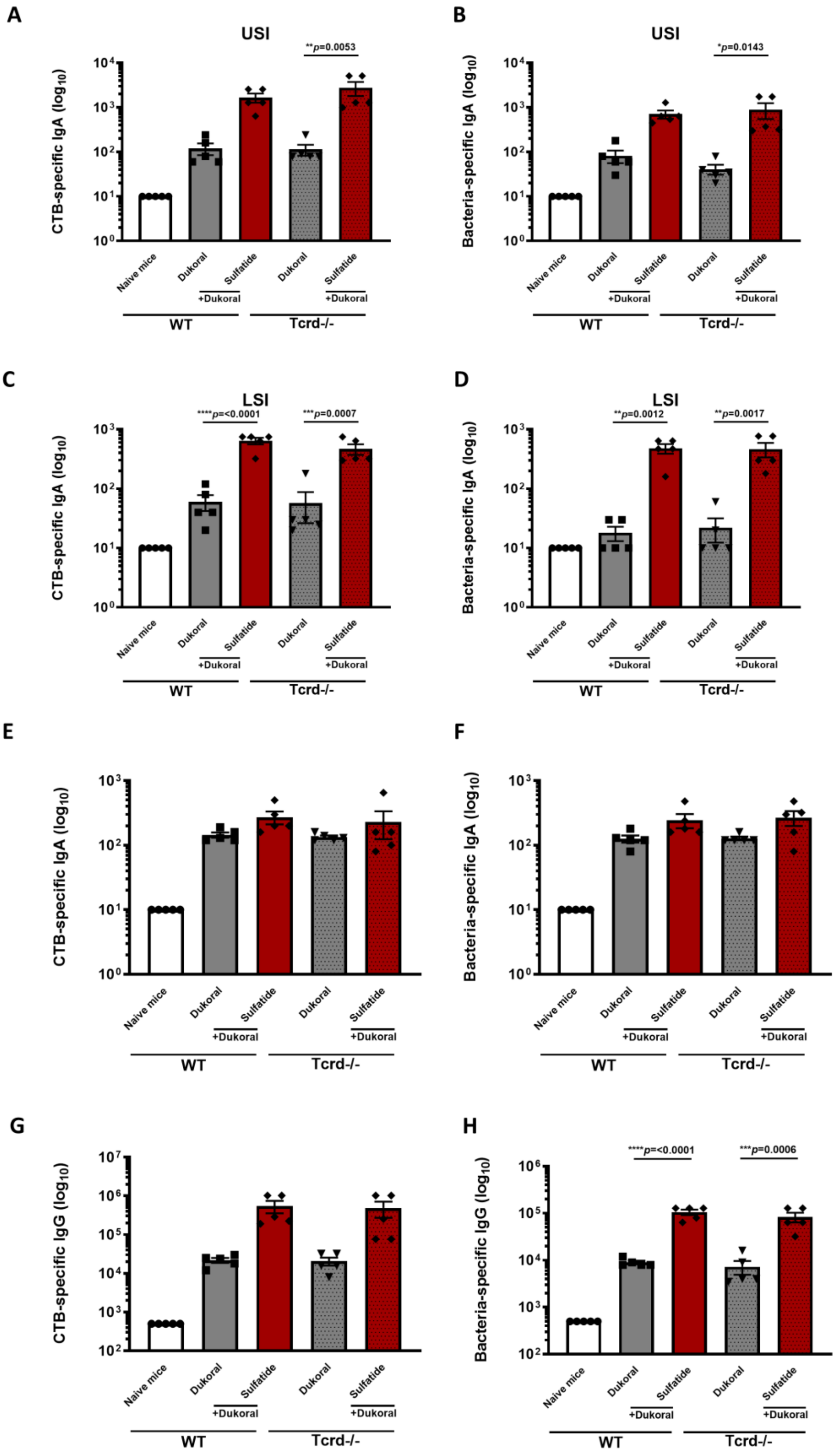

Figure S2. A similar increase in antigen-specific IgA and IgG responses is measured in WT and Tcrd-/- mice following oral co-administration with Dukoral® and sulfatide. Symbols show the groups of mice. Circles: naïve mice.

Squares: mice immunised with Dukoral® alone. Rhombus: mice immunised with Dukoral® and sulfatide. Each symbol represents one animal in the group.
